# Cloning of Nitrate Reductase and Nitrite Reductase Genes and Their Functional Analysis in Regulating Cr(VI) Reduction in Ectomycorrhizal Fungus *Pisolithus* sp.1

**DOI:** 10.3389/fmicb.2022.926748

**Published:** 2022-07-07

**Authors:** Liang Shi, Binhao Liu, Xinzhe Zhang, Yuan Bu, Zhenguo Shen, Jianwen Zou, Yahua Chen

**Affiliations:** ^1^College of Life Sciences, Nanjing Agricultural University, Nanjing, China; ^2^College of Resources and Environmental Sciences, Nanjing Agricultural University, Nanjing, China; ^3^Jiangsu Collaborative Innovation Center for Solid Organic Waste Resource, Nanjing Agricultural University, Nanjing, China; ^4^National Joint Local Engineering Research Center for Rural Land Resources Use and Consolidation, Nanjing Agricultural University, Nanjing, China; ^5^The Collaborated Laboratory of Plant Molecular Ecology (between College of Life Sciences of Nanjing Agricultural University and Asian Natural Environmental Science Center of the University of Tokyo), Nanjing Agricultural University, Nanjing, China

**Keywords:** Cr(VI) reduction, ectomycorrhizal fungi, nitrate reductase, phytoremediation, tolerance, yeast

## Abstract

Assimilatory-type nitrate reductase (NR) and nitrite reductase (NiR) are the key enzymes that involve in nitrate assimilation and nitrogen cycling in microorganisms. NR and NiR with NADH or NADPH and FMN or FAD domains could be coupled to the reduction process of hexavalent chromium [Cr(VI)] in microorganisms. A new assimilatory-type NR gene (named *niaD*) and a new assimilatory-type NiR gene (named *niiA*) are cloned, identified, and functionally characterized by 5′ and 3′ RACE, alignment, annotation, phylogenetic tree, and yeast mutant complementation analyses from *Pisolithus* sp.1, a dominant symbiotic ectomycorrhizal fungi (EMF) that can assist in phytoremediation. Assimilatory-type *niaD* and *niiA* were 2,754 bp and 3,468 bp and encode a polypeptide with 917 and 1,155 amino acid residues, respectively. The isoelectric points of NR (*Pisolithus* sp.1 NR) and NiR (*Pisolithus* sp.1 NiR) of *Pisolithus* sp.1 are 6.07 and 6.38, respectively. The calculated molecular mass of *Pisolithus* sp.1 NR and *Pisolithus* sp.1 NiR is 102.065 and 126.914 kDa, respectively. Yeast mutant complementation analysis, protein purification, and activities of NR and NiR under Cr treatment suggest that *Pisolithus* sp.1 NR is a functional NR that mediates Cr(VI) tolerance and reduction. The multiple alignment demonstrates that *Pisolithus* sp.1 NR is potentially a nicotinamide adenine dinucleotide phosphate-dependent flavin mononucleotide reductase and also Class II chromate reductase. Our results suggest that *Pisolithus* sp.1 NR plays a key role in Cr(VI) reduction in the EMF *Pisolithus* sp.1.

## Introduction

Chromium (Cr) is one of the top 20 pollutants in the list of super hazardous substances all over the world (Johnston and Chrysochoou, 2012; Dhal et al., 2013). The toxicity of hexavalent chromium [Cr(VI)] is 100 times that of trivalent chromium [Cr(III)], and the toxicity can be related to the solubility. Cr(VI) is highly soluble and toxic to microorganisms, plants, and animals, entailing mutagenic and carcinogenic effects whereas the latter is considered to be less soluble and less toxic (Nancharaiah et al., 2010). Excessive Cr(VI) and Cr(III) can cause lung cancer, kidney disease, dermatitis, etc. (Das et al., 2014; Viti et al., 2014). Therefore, the reduction of Cr(VI) to Cr(III) constitutes a potential detoxification process that might be achieved chemically or biologically.

Ectomycorrhizal fungi (EMF) can help host plants absorb water and mineral elements, improve the survival and growth rate of the host plants, and beneficial for their surviving in a variety of harsh and barren environments such as pests and diseases, salt stress, heavy metal (HM) stress, and drought stress (Pena and Polle, 2013; Chen Y.H. et al., 2015; Wen et al., 2017; Shi et al., 2018, 2019). *Pisolithus* sp. as one kind of EMF is widely distributed all over the world and can tolerate HMs such as manganese (Mn), copper (Cu), and lead. In addition, *Pisolithus* sp. can enhance the Cu tolerance of host plants such as *Acacia, Eucalyptus urophylla*, and black pine (Aggangan and Aggangan, 2012; Silva et al., 2013; Wen et al., 2017) while can also significantly reduce the stress of Mn on *Eucalyptus grandis* (Canton et al., 2016). In the previous study, we collected, isolated, and screened a strain of *Pisolithus* sp.1 (accession number: KY075875.1) with the ability of Cr(VI) reduction. In liquid medium, 75% of Cr(VI) can be reduced to Cr(III), and extracellular reduction of Cr(VI) can be accelerated by hydrogen ions (H^+^) generated by *Pisolithus* sp.1 by reducing the pH in the medium within 12 days (Shi et al., 2018). In addition, through the transcriptome sequencing of *Pisolithus* sp.1 [Cr(VI)-tolerant strain] and *Pisolithus* sp.2 [Cr(VI)-sensitive strain] before and after Cr(VI) treatment, the results of comparative transcriptome analysis found that compared with the control group [without Cr(VI)], the differentially expressed genes in *Pisolithus* sp.1 were only significantly enriched in the nitrogen metabolism pathway but did not significantly enrich in *Pisolithus* sp.2, and the relative expression levels of the genes encoding nitrate reductase (NR) (*niaD*) and nitrite reductase (NiR) (*niiA*) in *Pisolithus* sp.1 were higher in the presence of 10 mg/L Cr(VI) treatment (Shi et al., 2020).

Cr(VI) can interfere with nitrogen metabolism in plants, and NR plays an important role in the response of plants to Cr (Singh et al., 2013). For example, selenium nanoparticles can induce and increase the activity of NR in wheat, which can regulate Cr(VI) reduction (Yu et al., 2016). The expression fold of genes encoding the dissimilatory NR (narIJHG) and napAB, and the assimilation NR nasA, can be upregulated 3–20 times after been treated by 10 mg/L Cr(VI). In addition, the denitrification genes encoding the NiR NirK, the NO reductase NorB and the N_2_O reductase NosZ were upregulated 67, 152 and 207.5 times, respectively, under Cr(VI) treatment (Viamajala et al., 2002; Han et al., 2010). The above results showed that Cr(VI) has a positive effect on the expression of assimilative or dissimilatory genes encoding NR and NiR. Studies have also found that Cr(VI) can interact with various oxidoreductase in bacteria, including iron reductase, NR, NiR, glutathione reductase, lipid-based reductase, ferrioxy acid-NADP^+^ reductase, and other metal reductase while to be reduced by themselves (Ahemad, 2014). Therefore, the oxidation state of Cr [such as Cr(VI)] may induce some oxidoreductases to participate in the redox process, and this process will be coupled with the reduction of Cr(VI) to Cr(III) (Chovanec et al., 2012).

Actually, NR and NiR genes involved in nitrate assimilation are completely distinct from reductases involved in denitrification (dissimilation), which is a respiratory system. Assimilative nitrate reduction is a process in which nitrate is reduced to nitrite and ammonia, and ammonia is assimilated into amino acids. The reduced nitrogen here becomes the nitrogen source for the microorganisms. The dissimilatory nitrate reduction is nitrate respiration by microorganisms under anaerobic or micro-oxygen conditions, that is, NO${}_{3}^{-}$ or NO${}_{2}^{-}$ is used instead of O_2_ as an electron acceptor for respiratory metabolism. Dissimilatory nitrate reduction is further divided into fermentative nitrate reduction and respiratory nitrate reduction. In fermentative nitrate reduction, nitrate is an “incidental” electron acceptor in the fermentation process, rather than a terminal acceptor, which is an incomplete reduction, and the fermentation products are mainly nitrite and NH${}_{4}^{+}$. The products of respiratory nitrate reduction are gaseous N_2_O, N_2_, and this process is called denitrification (Il’ina and Khodakova, 1973; Jin et al., 2019). At present, there is no information about the type of genes encoding NR and NiR in *Pisolithus* sp., and NR or NiR has not been reported to be involved in Cr(VI) reduction in EMF. To solve the above questions, in this study, assimilatory NR and NiR genes were cloned, identified, analyzed, and functionally characterized from *Pisolithus* sp.1 by 5′ and 3′ RACE, alignment, yeast mutant complementation analysis, protein purification, and its reducing ability to Cr(VI).

## Materials and Methods

### Strains, Plasmids, and Culture Conditions

*Pisolithus* sp.1 (KY075875) was isolated from a sporophore in Sanqing Mountain, Jiangxi Province, China (28.54°N, 118.03°E), and the detailed information was referred to Shi et al. (2018). The mycelia of *Pisolithus* sp.1 were incubated in solid Kottke medium at 25°C for 18 days, and then, mycelia were collected, quickly frozen in liquid nitrogen, and saved at –80°C for RNA extraction and gene cloning. *Escherichia coli* DH5α was used for the transformation and propagation of plasmids and was cultured in Luriae–Bertani (LB) medium at 37°C. Cr(VI) tolerance assays were performed using the wild-type (WT) *Saccharomyces cerevisiae* strain BY4741 (MATa *his3*Δ*1 leu2*Δ*0 met15*Δ*0 ura3*Δ*0*) and Cr-sensitive Δ*ycf1* mutant (MATa *his3*Δ*1 leu2*Δ*0 met15*Δ*0 ura3*Δ*0 YCF1:kanMXX4*), which were grown in both yeast extract peptone dextrose medium and a synthetic defined medium at 30°C. The pYES2-NTB vector was used for heterogenous expression of the *niaD* and *niiA* gene in *S. cerevisiae.*

### RNA Extraction and cDNA Synthesis

For total RNA extraction, approximately 0.5 g of fresh mycelia from the *Pisolithus* sp.1 strain was frozen, grounded into a fine powder in liquid nitrogen and homogenized in RNAiso Plus solution. Total RNAs were extracted using the RNAiso Plus Kit (TaKaRa, Dalian, China) according to the manufacturer’s protocol. Then, the quantity and the purity of the RNA were determined by UV measurement using the NanoDrop 2000c spectrophotometer (Thermo Scientific, Shanghai, China). First-strand cDNA was synthesized from the total RNA using the PrimeScriptII Frist-Strand cDNA Synthesis Kit (TaKaRa, Dalian, China). The synthesized first-strand cDNA was used as the PCR template and saved at –20°C.

### Cloning of *niaD* and *niiA*

Based on the results of transcriptome sequencing (Shi et al., 2020; accession number: SRR8837356-SRR8837367), compared with the control group [without Cr(VI)], the differentially expressed genes in *Pisolithus* sp.1 were only significantly enriched in the nitrogen metabolism pathway but did not significantly enrich in *Pisolithus* sp.2, and the relative expression levels of the NR gene *niaD* and NiR gene *niiA* in *Pisolithus* sp.1 were higher in the presence of 10 mg/L Cr(VI) treatment. The 5′ race and 3′ race primers of *niaD and niiA* genes were designed based on the sequencing results of *niaD* and *niiA* (Table 1 and Supplementary Table 1). For 5′ race PCR amplification, using cDNA of *Pisolithus* sp.1 as a template, PCR system (25 μl): template cDNA 0.5 μl, 5 GeneRacer outer primer (10 μm) 1.0 μl, *niaD*-R1 or *niiA*-R1 (10 μm) 1.0 μl, and Platinum PCR SuperMix High Fidelity (Invitrogen) 22.5 μl. The first round of PCR condition is pre-denaturation at 94°C for 2 min; denaturation at 94°C for 30 s, annealing and extension at 72°C for 30 s, 5 cycles; then denaturation at 94°C for 30 s, annealing and extension at 70°C for 30 s, 5 cycles; and denaturation at 94°C for 30 s, annealing and extension at 66°C for 30 s, 25 cycles. For the second round of PCR, using the production of the first-round PCR as a template, PCR system (25 μl): template cDNA 0.5 μl, 5 GeneRacer Inner primer (10 μm) 1.0 μl, *niaD*-R2 or *niiA*-R2 (10 μm) 1.0 μl, 10 Platinum PCR SuperMix High Fidelity (Invitrogen) 22.5 μl. The second round of reaction conditions is pre-denaturation at 94°C for 2 min; denaturation at 94°C for 30 s, annealing and extension at 66°C for 30 s, 30 cycles. For 3′ race PCR amplification, the first and the second reaction condition and system are the same with 5′race, just use 3GeneRacer Inner/Outer primer, *niaD*-F1, *niaD*-F2, *niiA*-F1, *niiA*-F2 instead of 5GeneRacer inner/outer primer, *niaD*-R1, *niaD*-R2, *niiA*-R1, and *niiA*-R2, respectively. After 5′ and 3′ RACE, the PCR products were purified using the BioTeke Gel Extraction Kit (BioTeke, Beijing, China). The fragment was cloned into the pMD18-T Simple Cloning Vector (TaKaRa, Dalian, China) and transformed into competent *Escherichia coli* DH5a for DNA sequencing. To obtain the complete *niaD* and *niiA*, full-length PCR was performed with high fidelity KOD FX (Toyobo, Shanghai, China) using the primers *niaD* full length and *niiA* full length (Table 1).

**TABLE 1:** Primers used in this study.

Gene name	Primer name	Sequence (5′-3′)
*niaD-*5′race	*niaD-R1*	CAATTGTCAGGAGTCTCGCGATCCA
	*niaD-R2*	CGTCGTGGAGAGATGCTGGTAAGAATG
	Outer primer	CGACTGGAGCACGAGGACACTGA
	Inner primer	GGACACTGACATGGACTGAAGGAGTA
*niiA-*5′race	*niiA-R1*	GCCGAGCGTACCATTCCACTTGAT
	*niiA-R2*	GCGATTGTAAGCCAGGTGCGTTT
	Outer primer	CGACTGGAGCACGAGGACACTGA
	Inner primer	GGACACTGACATGGACTGAAGGAGTA
*niaD-*3′race	*niaD–F1*	GACACCAGCGATACAGAGACGAGAGT
	*niaD–F2*	GGAGGACATTCTCTGTCGTGCTGAACT
	Outer primer	GCTGTCAACGATACGCTACGTAACG
	Inner primer	CGCTACGTAACGGCATGACAGTG
*niiA-*3′race	*niiA–F1*	CGGTACCTGCCTGAACGACATGCA
	*niiA–F2*	GGGACGACCTGCAGCTCCTGCTT
	Outer primer	GCTGTCAACGATACGCTACGTAACG
	Inner primer	CGCTACGTAACGGCATGACAGTG
*niaD-*full length	Forward primer	AAGATATCTTAAGAATGTTTGACG
	Reverse primer	CCTATACAGCAAACACAGGCAGAT
*niiA-*full length	Forward primer	GACTCGGTTGGAGCCTTATC
	Reverse primer	CAATCTGACTGCTCAATGCATACGA
*niaD-*Yeast	Forward primer	^a^ACCGAGCTCGGATCCATGT
complementation		TTGACGAATATGTCGAC
	Reverse primer	^b^ATGCGGCCCTCTAGAT
		CAGAATACTACGAGGTGGT
*niiA-*Yeast	Forward primer	^a^ACCGAGCTCGGATCCA
complementation		TGATGAACAGTACACTAGGC
	Reverse primer	^b^ATGCGGCCCTCTAG
		ATCAGGCAGGTACCGTCGCTA

*Underlined sequences are a, BamH1 and b, Xba1 sites.*

### Bioinformatic Analysis

The open reading frame (ORF) was predicted using the ORF Finder^1^. The cloned *niaD* and *niiA* were analyzed to predict the amino acid sequences using the DNAMAN software package (version 7.0.2.176, Lynnon BioSoft, Canada). The amino acid sequences of *niaD* and *niiA* were analyzed and performed using protein Basic Local Alignment Search Tool (BLAST) algorithms^2^. The predicted amino acid sequences were used to search for conserved domains with NCBI Conserved Domain Search database^3^. The theoretical pIs and molecular masses were predicted using the Compute pI/Mw tool from the Expert Protein Analysis System (ExPASy) database^4^). The potential transmembrane domains in the protein sequences were predicted using the TMHMM Server v.2.0^5^, HMMTOP program^6^ (Tusnády and Simon, 2001), TMpred^7^, and the network protein sequence analysis (NPSA)^8^. The putative amino acid sequences of *niaD* and *niiA* and the other NRs and NiRs from different organisms were aligned using the DNAMAN software package and the Clustal X program, version 1.83. A phylogenetic analysis was performed using the maximum likelihood method in the MEGA5 program.

### Gene Function Analysis

To insert the *niaD* or *niiA* gene into the expression vector pYES2, the cloned genes were amplified by PCR using primers with *BamH1* and *Xba1* restriction sites (Table 1). The amplified product was cloned into the corresponding restriction site of pYES2 vector, and the yeast cells were transformed by electro-transformation after sequencing verification (Rono et al., 2021). The transformed yeast cells were selectively cultured on SD-Ura solid medium which containing 2% glucose (mass/volume ratio) but the lack of uracil, and clones were picked after 2–3 days. The plasmids were extracted for PCR verification, and the recombinant strain was stored at –70°C. The mutant strain (Δ*ycf1*), which was transformed using the empty pYES2 vector, was used as controls. To determine the Cr(VI) tolerance of recombinant yeast cells, receptively, the recombinant yeast strains (Δ*ycf1*-pYES2, Δ*ycf1-*pYES2-*niaD*, and Δ*ycf1-*pYES2-*niiA*) were cultured on SD-Ura liquid medium which containing 2% glucose to optical density (OD_600_) = 1.0. All the cultures were serially diluted (10^0^, 10^–1^, 10^–2^, 10^–3^, 10^–4^, and 10^–5^) and 5 μl of each dilution was spotted on SD-Ura solid medium with and without Cr (0, 10, 20, and 40 mg L^–1^). The spotted plates were then incubated at 30°C for 3 days and photographed.

Further, the tolerance and reduction abilities of transformants to Cr(VI) were also measured in a liquid SD broth. The transformants Δ*ycf1*-pYES2, Δ*ycf1-*pYES2-*niaD* and Δ*ycf1-*pYES2-*niiA* were inoculated separately in 50 ml SD-Ura liquid medium with the starting OD_600_ of 0.05 and allowed to grow at 30°C for 5 h at 220 rpm. After 5 h, the cultures were subjected to different concentrations of Cr(VI) (0, 5, and 10 mg/L) and incubated at 30°C for 24 h. The effect of Cr(VI) on each culture was recorded as OD_600_ after 24 h, and compared with control [without Cr(VI)], we calculate the inhibition rate of each strain in 5 and 10 mg/L of Cr(VI) treatments. In addition, we determine Cr(VI) concentration in supernatant of all treatments and calculate the Cr(VI) reduction rates of each strain. For Cr determination, after samples were treated by Cr(VI) for 24 h, the supernatant of samples was obtained by the centrifugation at 4,000 rpm for 10 min and was passed through a 0.22-μm filter. Cr was analyzed by atomic absorption spectrometry (ZEEnit700^®^ P, Analytik Jena AG, Germany). About 1 ml aliquots of medium were passed through 0.22-μm filters, and 1,000 μl of 1,5-diphenylcarbazide (0.5 g in 100 ml absolute ethanol and 400 ml 3.6 N H_2_SO_4_) was added. The violet complex formed was analyzed at 540 nm using a UV–vis spectrophotometer (UV2450, Shimadzu Business Systems Corporation, Japan). The percentage of Cr (VI) reduction was calculated using the following formulas:


$$C_{PR}=\left(A_{oCr\left(VI\right)}-A_{sCr\left(VI\right)}\right)/A_{oCr\left(VI\right)}\times 100$$

where *C*_*PR*_ is the percentage of Cr(VI) reduction, *A*_*oCr(VI)*_ is the original absorbance of the samples which are treated by Cr for 0 h after inoculation with yeast, and *A*_*sCr(VI)*_ is the absorbance of the samples which are treated by Cr for 24 h after inoculation with yeast.

### Protein Purification and Activities

The *niaD* and *niiA* genes were inserted into vector pET28b, creating plasmid pET28b-*niaD* and pET28b-*niiA*. Plasmids pET28b-*niaD* and pET28b-*niiA* were transformed into *Escherichia coli* (*E. coli*) BL21 and grown aerobically in 100 ml LB medium supplemented with 50 μg ml^–1^ kanamycin at 37°C to OD_600_ = 0.5, at which time 0.3 mm isopropyl β-D-1-thiogalactopyranosid (IPTG) was added, and growth was continued at 37°C for an additional 4 h. The cells were harvested by centrifugation (15 min at 5,000 × *g*), and NR was purified as described previously (Chen J. et al., 2015). Protein purity was confirmed by sodium dodecyl sulfate polyacrylamide gel electrophoresis (SDS-PAGE), and protein concentrations were determined using the Bradford method (Bradford, 1976).

NR and NiR activities were determined at 37°C in an assay mixture containing 5 μm Cr(VI) in 1 ml of 25 mm of Bis-Tris Propane buffer (pH 7.0). Purified NR/NiR (1 μm), 0.2 mM NADPH or NADH, and 25 μm FMN or FAD were added. At 0–160 min, 0.1 ml portions were filtered through 3-kDa cutoff Amicon Ultrafilters (Millipore). Cr(VI), Cr(III), and total Cr in the filtrate were determined referred to Shi et al. (2018).

### Statistical Analysis

The data were analyzed using analysis of variance (ANOVA) (SPSS 16.0; SPSS, Inc., Chicago, IL, United States), followed by Tukey’s honestly significant difference (HSD) test (*p* < 0.05) to determine the differences between the yeast strains according to each treatment. The data represent the mean ± standard deviation (SD) for four independent replicates.

## Results

### Cloning of *niaD* and *niiA*

**FIGURE 1:**
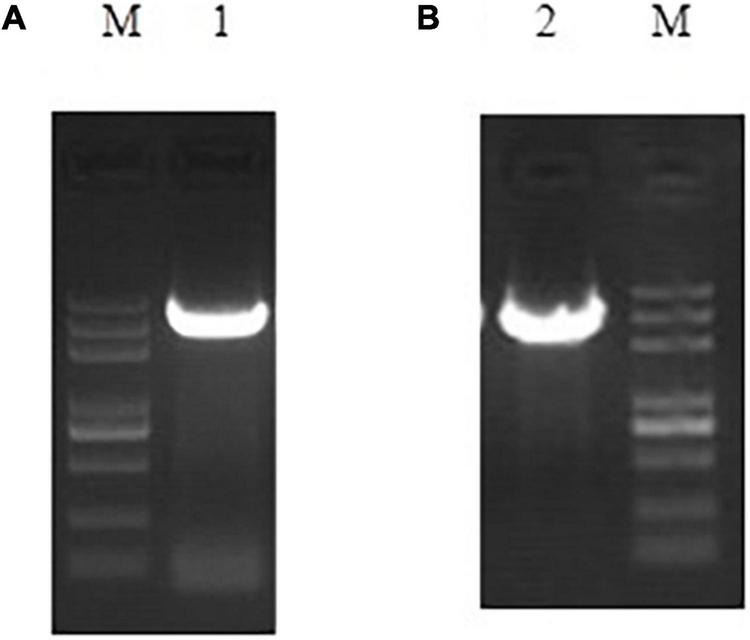
Full-length PCR amplification results of *niiA* **(A)** and *niaD* **(B)**.

Using the 5′ RACE (Supplementary Figure 1) and 3′ RACE (Supplementary Figure 2) techniques, we cloned the full-length cDNA sequences of the gene encoding the assimilatory nitrate reductase (NR) (*Pisolithus* sp.1 NR) named *niaD* with 2,932 bp (GenBank accession number: OM220115) and the gene encoding the assimilatory nitrite reductase (NiR) (*Pisolithus* sp.1 NiR) named *niiA* with 3,982 bp (GenBank accession number: OM220116) length (Figure 1). The open reading frame (ORF) of *niaD* consisted of 2,754 bp predicted to code 917 amino acids (GenBank accession number: OM339169), and the ORF of *niiA* with 3,468 bp length corresponded to 1,155 amino acids (GenBank accession number: OM339170). The isoelectric point (pI) of *Pisolithus* sp.1 NR and the calculated molecular mass were 6.07 and 102.065 kDa, respectively; the pI of *Pisolithus* sp.1 NiR and the calculated molecular mass were 6.38 and 126.914 kDa, respectively. Based on the amino acid sequences of two proteins, we found 51.4% of hydrophobic and 48.4% of hydrophilic amino acids in the *Pisolithus* sp.1 NR whereas 48.2% of hydrophobic and 51.9% of hydrophilic amino acids in the *Pisolithus* sp.1 NiR. The ExPASy online hydrophobic predications of these two proteins are also shown in Supplementary Figure 3. The results from the NCBI Conserved Domain Search further confirmed that *Pisolithus* sp.1 NR and *Pisolithu*s sp.1 NiR belong to the PLN02252 and Rieske superfamily, respectively. However, there were no any transmembrane-spanning (TMs) domains found in *Pisolithus* sp.1 NR and *Pisolithus* sp.1 NiR using TMHMM software (Supplementary Figure 4). The signal peptide prediction analysis indicated that no signal peptide sequence was observed in *Pisolithus* sp.1 NR or *Pisolithus* sp.1 NiR.

### Phylogenetic Analysis of Protein

The deduced *Pisolithus* sp.1 NR proteins were compared with other 18 EMF NRs, and *Pisolithus* sp.1 NiR proteins were compared with other 40 NiRs of EMF (the species name, gene IDs, and accession number are shown in Supplementary Tables 2, 3. A total of two main clades were distinguished (Supplementary Figure 5A), one branch for the *Pisolithus* sp.1 NR, *Pisolithus tinctorius* NR and another 16 EMF NRs, and the second branch for the *Coprinopsis cinerea okayama* NR. *Pisolithus* sp.1 NR was closely related to *Pisolithus tinctorius* NR (AGO04408, 100% similarity). For NiR, two main clades were distinguished (Supplementary Figure 5B), one branch for the *Pisolithus* sp.1 NiR, *Paxillus ammoniavirescens* NiR and another 15 EMF NiRs, and the second branch for the *Polyporus arcularius* NiR, *Polyporus brumalis* NiR and another 22 EMF NiRs. *Pisolithus* sp.1 NiR was closely related to *Paxillus ammonia virescens* NiR (KAF8843892, 99% similarity).

### Multiple Sequence Alignment

The homology comparison results of *Pisolithus* sp.1 NR ORF BlastP showed that the top 18 EMF with the highest homology to the amino acid sequence encoded by *Pisolithus* sp.1 NR are *Pisolithus tinctorius* (AGO04408.1), *Paxillus involutus* (KIJ15906.1), *Suillus tomentosus* (KAG1876842.1), and so on (Supplementary Figure 6). After multiple comparisons of the amino acid sequence encoded by *Pisolithus* sp.1 NR with the above-mentioned 18 fungal sequences, it is found that there is high homology between these 19 amino acid sequences, especially in the conserved binding domains that constitute NR, such as molyopterin binding domains, which the homology is the highest (Supplementary Figure 6). The above results indicate that the full-length sequence of *niaD* cloned by RACE is the complete sequence of the NR gene.

**FIGURE 2:**
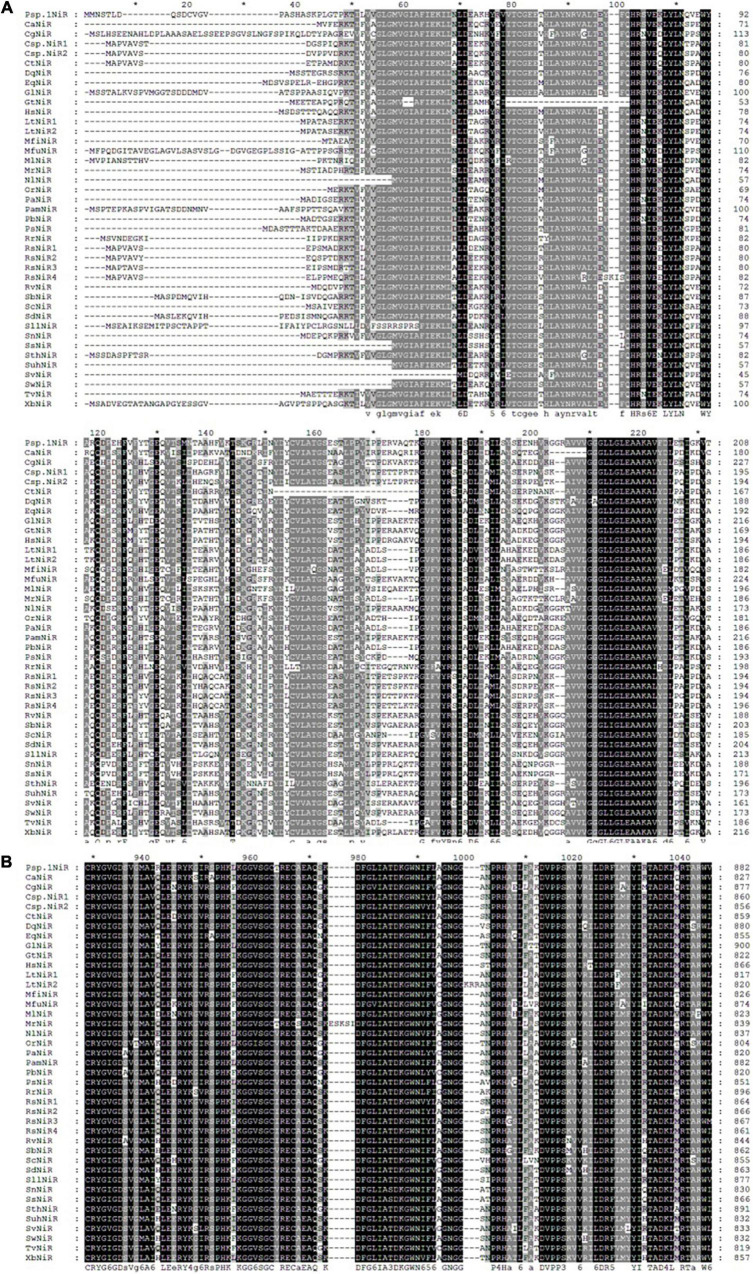
Multiple sequence alignment of the N-terminal part **(A)** and the region corresponding to Fe/S and siroheme domain **(B)** in *niiA* encoded amino acid from *Pisolithus* sp.1 and other EMF. The two signatures, CXXXXXC and CXXXC, that characterize the (Fe/S)-siroheme-binding site are indicated (I, II). The GXGXXG motifs probably involved in nucleotide binding. The two signatures, CXXXXXC and CXXXC, that characterize the (Fe/S)-siroheme-binding site are indicated (I, II). Accession numbers are as follows: *Pisolithus* sp.1 (Pisolithus sp.1NiR); *Cantharellus anzutake* (CaNiR), XP_038913312; *Cortinarius glaucopus* (CgNiR), KAF8804920; *Ceratobasidium* sp. (Csp.NiR1), KAF8599087; *Ceratobasidium* sp. (Csp.NiR2), QRW14243; *Ceratobasidium theobromae* (CtNiR), KAB5593146; *Daedalea quercina* (DqNiR), KZT68487; *Exidia qlandulosa* (EqNiR), KZV93274; *Gyrodon lividus* (GlNiR), KAF9219484; *Gloeophyllum trabeum* (GtNiR), XP_007868164; *Heliocybe sulcata* (HsNiR), TFK51421; *Lentinus tigrinus* (LtNiR1), RPD71067; *Lentinus tigrinus* (LtNiR2), RPD56290; *Marasmius fiardii* (MfiNiR), KAF9267857; *Macrolepiota fuliginosa* (MfuNiR), KAF9446723; *Microbotryum lychnidis-dioicae* (MlNiR), KDE09322; *Moniliophthora roreri* (MrNiR), ESK92732; *Neolentinus lepideus* (NlNiR), KZT30290; *Obba rivulosa* (OrNiR), OCH89826; *Polyporus arcularius* (PaNiR), TFK84168; *Paxillus ammoniavirescens* (PamNiR), KAF8843892; *Polyporus brumalis* (PbNiR), RDX45056; *Punctularia strigosozonata* (PsNiR), XP_007380627; *Ramaria rubella* (RrNiR), KAF8587207; *Rhizoctonia solani* (RsNiR1), QRW27772; *Rhizoctonia solani* (RsNiR2), CEL51481; *Rhizoctonia solani* (RsNR3), CUA70758; *Rhizoctonia solani* (RsNR4), EUC59403; *Rhizopogon vinicolor* (RvNiR), OAX35873; *Suillus brevipes* (SbNiR), KAG3229875; *Sparassis crispa* (ScNiR), XP_027609679; *Suillus decipiens* (SdNiR), KAG2067852; *Suillus hirtellus* (SuhNiR), KAG2059534; *Stereum hirsutum* (SthNiR), XP_007309002; *Serpula lacrymans* var. *lacrymans* (SllNiR), XP_007318270; *Sistotremastrum niveocremeum* (SnNiR), KZS88669; *Sistotremastrum suecicum* (SsNiR), KZT38055; *Serendipita vermifera* (SvNiR), PVF95350; *Suillus weaverae* (SwNiR), KAG2343131; *Trametes versicolor* (TvNiR), XP_008033405; *Xerocomus badius* (XbNiR), KAF8552377.

The homology comparison results of *Pisolithus* sp.1 NiR ORF BlastP showed that the top 36 EMF with the highest homology to the amino acid sequence encoded by *Pisolithus* sp.1 NiR are *Xerocomus badius* (KAF8552377.1), *Gyrodon lividus* (KAF9219484.1), *Paxillus ammoniavirescens* (KAF8843892.1), *Heliocybe sulcata* (TFK51421.1), and so on (Figure 2). After multiple comparisons of the amino acid sequence encoded by *Pisolithus* sp.1 NiR with the above-mentioned 36 fungal sequences, it is found that there is high homology between these 37 amino acid sequences. The putative iron-sulfur center [4Fe-4S] and siroheme domains were localized in the predicted *Pisolithus* sp.1 NiR sequence by comparison with known NiRs. CX5CXnGCX3C as the consensus sequence, where the cysteine residues have been proposed to be involved in the binding of the tetranuclear iron-sulfur center and siroheme to the NiR. The 5 amino acids VGTTW separating the very N-terminus cysteines are identical in *P. ammoniavirescens, H. sulcata, Rhizopogon vinicolor*, and so on. The region around the cysteine consensus sequence is highly conserved among NiRs but poorly conserved between NiRs and sulfite reductase (Figure 2B). Regarding the FAD and NAD(P)H-binding domains, the amino acid sequence analyses revealed a common motif sequence GXGXXG compatible with a β sheet-α helix-β sheet folding. This motif has been found two times in the N-termini of NiRs although in *Suillus weaverae, Suillus hirtellus, Serpula lacrymans* var. *Lacrymans, Neolentinus lepideus, Gloeophyllum trabeum*, and *Sistotremastrum suecicum*, the Wrst is less conserved (Figure 2A).

### Functional Complementation Verification

**FIGURE 3:**
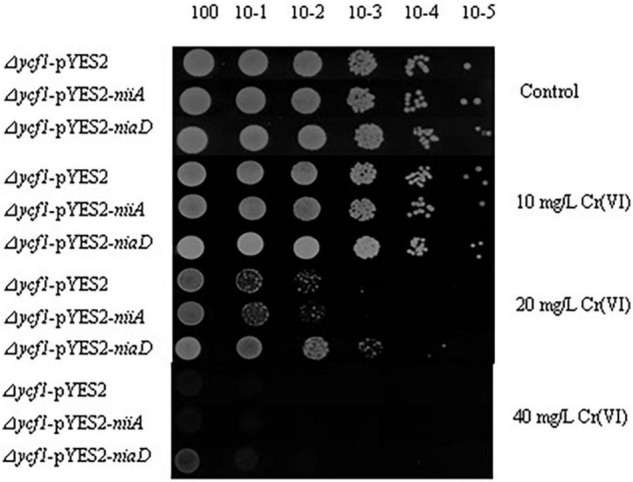
Functional complementation of Cr(VI)-sensitive yeast mutants on selective media. Mutant strains (Δ*ycf1*) were transformed with empty vector pYES2, pYES2-*niiA* or pYES2-*niaD*. Diluted transformant cultures were spotted on SD-Ura medium with or without metal supplement as indicated.

*niaD* and *niiA* were transformed into the yeast mutant (Δ*ycf1*) using the pYES2 vector to examine their functions of Cr(VI) tolerance and reduction. With the gradually dilution of the yeast cells, the growth of Δ*ycf1*-pYES2, Δ*ycf1*-pYES2-*niaD*, and Δ*ycf1*-pYES2-*niiA* strain decreased in SD-Ura solid medium with different Cr(VI) concentrations (0, 10, 20, and 40 mg/L) (Figure 3). Compared with the strongly inhibited growth of *ycf1* yeast cells transformed with the empty pYES2 vector, *niaD* expression recovered the yeast growth in 20 and 40 mg/L Cr(VI) treatments (Figure 3).

**FIGURE 4:**
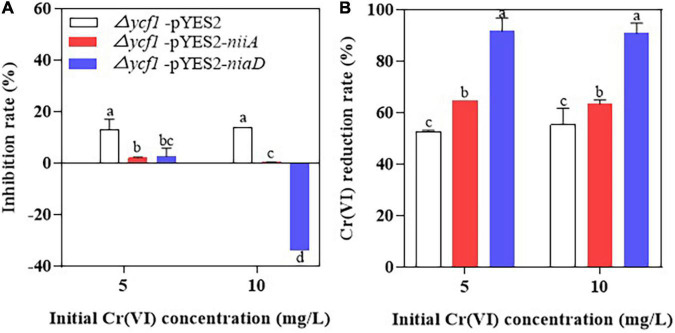
The inhibition rate of Cr(VI) on yeast growth **(A)** and Cr(VI) reduction rates of yeasts **(B)**. Mutant strains (Δ*ycf1*) were transformed with empty vector pYES2, pYES2-*niiA* or pYES2-*niaD* in SD-Ura liquid medium with or without metal supplementation.

It was also observed that the mutant strain (Δ*ycf1*) transformed with *niaD* and *niiA* grew well in Cr(VI)-supplemented SD-Ura liquid medium compared to the Δ*ycf1*-pYES2 strain, and 10 mg/L Cr(VI) did not inhibit the growth of Δ*ycf1*-pYES2-*niaD* strain compared with Δ*ycf1*-pYES2 and Δ*ycf1*-pYES2-*niiA* strains (Figure 4A). In addition, compared with Δ*ycf1*-pYES2 and Δ*ycf1*-pYES2-*niiA* strain, Δ*ycf1*-pYES2-*niaD* strain also has the highest Cr(VI) reduction ability (Figure 4B).

### Cr(VI) Reduction by Purified Protein

**FIGURE 5:**
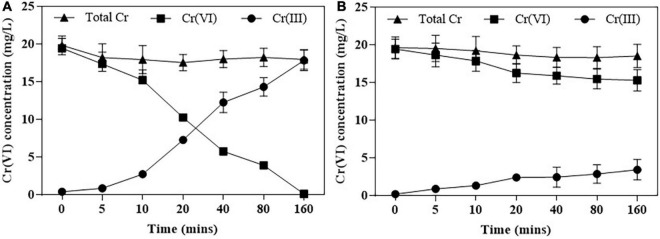
Cr(VI) reduction by purified NR **(A)** and NiR **(B)**. Cr(VI) reduction was assayed in Bis-Tris Propane buffer (pH 7.0). Error bars represent standard deviation (SD) from three independent assays.

The purification of *NR* and *NiR* was finished by SDS-PAGE (Supplementary Figure 7), Figure 5 shows that Cr(VI) was fully reduced by NR within 160 min, and Cr(VI) was 25% reduced by NiR at the same time.

## Discussion

The NR that exists in EMF is an enzyme composed of Mo element, flavin, and heme subunits. It participates in the assimilation of nitrate and provides N nutrition for mycorrhizal symbionts (Okiobe et al., 2019). The NR genes has been found in some EMF fungi, such as *Hebeloma* sp., *Tuber borchii, Laccaria bicolor*, and *Wilcoxina mikolae* var. m*ikolae*, which prove that NR plays an indispensable role in the establishment of EMF-plant symbiosis (Guescini et al., 2003).

In our study, the full-length cDNA of the NR and the NiR gene was cloned from the *Pisolithus* sp.1 using the 5′ and 3′ RACE approach, named *Pisolithus* sp.1 NR and *Pisolithus* sp.1 NiR, respectively. By comparing the amino acid sequence of NR with other 18 species of fungi, it is found that the homology between *Pisolithus* sp.1 NR and *Pisolithus tinctorius* NR reached up to 100%; the homology between *Pisolithus* sp.1 NiR and *Paxillus ammonia* virescens NiR up to 99%. *Pisolithus* sp.1 like some fungi, especially EMF fungi, has a conserved NR gene. Previous studies have proved that *Pisolithus* sp.1 can increase the N absorption of *Pinus thunbergii* (Shi et al., 2019). However, we did not find any transmembrane-spanning (TMs) domains and signal peptides in *Pisolithus* sp.1 NR and *Pisolithus* sp.1 NiR (Supplementary Figure 4), and they should belong to assimilatory enzymes.

The formation process of ectomycorrhiza involves the expression of genes related to symbiosis between plants and EMF. The expression of these genes at a specific time and space will cause the morphological and physiological changes, and this process plays an important role in the formation and coordinated development of mutually beneficial symbiosis (Quéré et al., 2005; Willmann et al., 2014).

On the other hand, *niaD* is the first NR gene to be identified and found in EMF with Cr(VI) tolerance and reduction function in our study. Some previous studies have shown that some NR or NiR have Cr(VI) reduction function in bacteria (Bencheikh-Latmani et al., 2005; Chai et al., 2018). For example, such NR of *Vibrio harveyi* KCTC 2720 and NfsA of *E. coli* have a facultative function to reduce Cr(VI) (Pradhan et al., 2016). It suggests that *niaD* is potentially a novel Cr(VI) reductase in EMF. In our previous study, the relative transcription of *niaD* significantly increased after *Pisolithus* sp.1 was treated by Cr(VI), which indicated that *niaD* was an Cr(VI)-inducible reducing enzyme (Shi et al., 2020).

**FIGURE 6:**
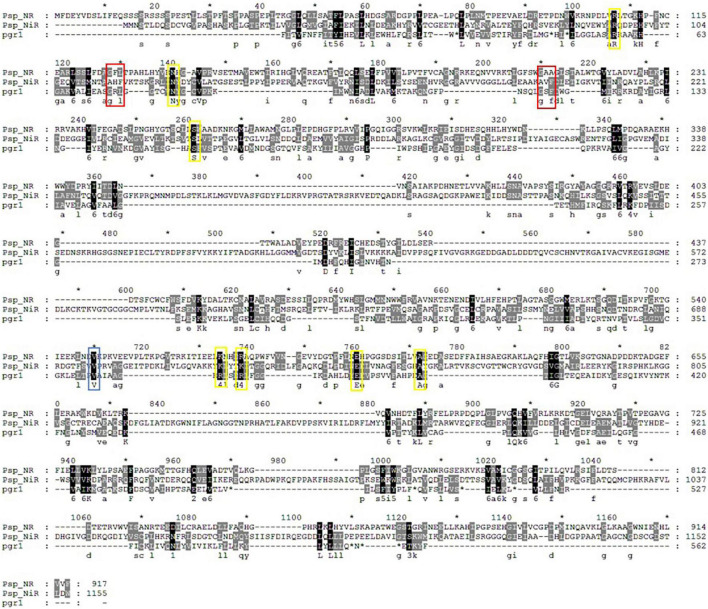
Multiple alignment of *Pisolithus* sp.1 NR, *Pisolithus* sp.1 NiR and the pgr1. The FMN-binding and NADPH-binding sites are labeled with yellow and blue rectangles, respectively, and the sites marked with red rectangles are both FMN- and NADPH-binding sites.

Chromate reductase includes Class I and Class II chromate reductase, and Class I chromate reductases are efficient chromate and quinone reducers, but have no activity with nitro compounds, whereas the Class II chromate reductase enzymes all reduce quinones and nitro compounds effectively, but vary in their ability to transform chromate (Pradhan et al., 2016). The Class I enzymes reduce chromate at a greater rate than the Class II enzymes (Park et al., 2002). As shown in Figure 6, *Pisolithus* sp.1 NR is highly conserved compared with pgr1 which was identified as a Class II chromate reductases (Koósz et al., 2008). The chromate reductases are also classified based on their homology and specificity for reducing Cr(VI). For example, the Class I chromate reductases transfer two electrons from themselves either simultaneously (“tight”; such as YieF) or non-simultaneously (“semi-tight”; such as ChrR) (Ackerley et al., 2004; Thatoi et al., 2014). NitR belongs to Class I chromate reductase and it bears poor homology with NfsA, one of the Class II chromate reductases. Therefore, *Pisolithus* sp.1 NR may share poor homology with Class I chromate reductase due to it belongs to Class II chromate reductase (Chai et al., 2018).

Various groups of oxidoreductase enzymes such as chromate reductase, NR, iron reductase, quinone reductase, hydrogenase, flavin reductase, and NAD(P)H-dependent reductase showing potential toward reduction of chromate have been identified in different microorganisms. In addition, comparative structural analyses of seven well-studied enzymes involved in chromate reduction from Protein Data Bank (PDB) database were categorized either NADPH-dependent FMN reductase or FMN-dependent NR (Pradhan et al., 2016). The FMN- and NADPH-binding sites are critical to the function of the chromate reductases. FMN is firmly anchored to the reductases by several hydrogen bonds and transfer the electron from NADH to the substrate scilicet Cr(VI) (Eswaramoorthy et al., 2012). Hence, FMN-binding site represents the enzyme active site (Matin et al., 2005) and hydrogen bonds between FMN molecules and different monomers possibly play a central role in chromate reductase activity. The NADPH-binding site is also important for enzyme activity, and NAD(P)H provides electrons for Cr(VI) reduction. The FMN- and NADPH-binding sites of *Pisolithus* sp.1 NR are more conserved with that of pgr1, all of which are identical or conserved substitutions (Figure 6). Combined with the results in Figure 5, 0.2 mm NADPH and 25 μm FMN were the best cofactor for the reduction of Cr(VI) by *Pisolithus* sp.1. Therefore, *Pisolithus* sp.1 NR is possibly a NADPH-dependent FMN-reductase, which reduces Cr(VI) with the identical electron transport pathway of pgr1.

In conclusion, *niaD* and *niiA* are novel NR and NiR genes in *Pisolithus* sp.1, were cloned, identified, and functionally characterized by 5′ and 3′ RACE, alignment, annotation, phylogenetic tree, yeast mutant complementation analysis, protein purification, and function on Cr(VI) reduction. The open reading frame (ORF) of *niaD* consisted of 2,754 bp predicted to code 917 amino acids, and the ORF of *niiA* with 3,468 bp length corresponded to 1,155 amino acids. *Pisolithus* sp.1 NR and *Pisolithus* sp.1 NiR had no transmembrane-spanning (TMs) domains. *Pisolithus* sp.1 NR is highly conserved compared with pgr1 (chromate reductase in *Schizosaccharomyces pombe*), which was identified as a Class II and NADPH-dependent FMN chromate reductase.

## Data Availability Statement

The datasets presented in this study can be found in online repositories. The names of the repository/repositories and accession number(s) can be found in the article/Supplementary Material.

## Author Contributions

LS contributed to the experimental design, conceptualization, data curation, and original draft. BL carried out the experiments and data analysis. XZ performed the data analysis. YB interpreted the data. ZS and JZ contributed to the review, supervision, and conceptualization. YC contributed to the supervision, conceptualization, writing, reviewing, and editing. All authors contributed to the article and approved the submitted version.

## Conflict of Interest

The authors declare that the research was conducted in the absence of any commercial or financial relationships that could be construed as a potential conflict of interest.

## Publisher’s Note

All claims expressed in this article are solely those of the authors and do not necessarily represent those of their affiliated organizations, or those of the publisher, the editors and the reviewers. Any product that may be evaluated in this article, or claim that may be made by its manufacturer, is not guaranteed or endorsed by the publisher.

## Footnotes

1
http://www.ncbi.nlm.nih.gov/gorf/orfig.cgi

2
http://www.ncbi.nlm.nih.gov/BLAST

3
http://www.ncbi.nlm.nih.gov/Structure/cdd/wrpsb.cgi

4
http://www.expasy.ch/tools/pi_tool.html

5
http://www.cbs.dtu.dk/services/TMHMM/

6
http://www.enzim.hu/hmmtop/

7
http://www.ch.embnet.org/software/TMPRED_form.html

8
http://npsa-pbil.ibcp.fr/cgi-bin/npsa_automat.pl?page?/NPSA/npsa_htm.html
