# Supplementary material for: Cloning of Nitrate Reductase and Nitrite Reductase Genes and Their Functional Analysis in Regulating Cr(VI) Reduction in Ectomycorrhizal Fungus Pisolithus sp.1
Source: Front Microbiol. 2022 Jul 7;13:926748. doi: 10.3389/fmicb.2022.926748 (PMC9301267; doi:10.3389/fmicb.2022.926748)
Supplement: Supplementary file 1 [file Data_Sheet_1.PDF]

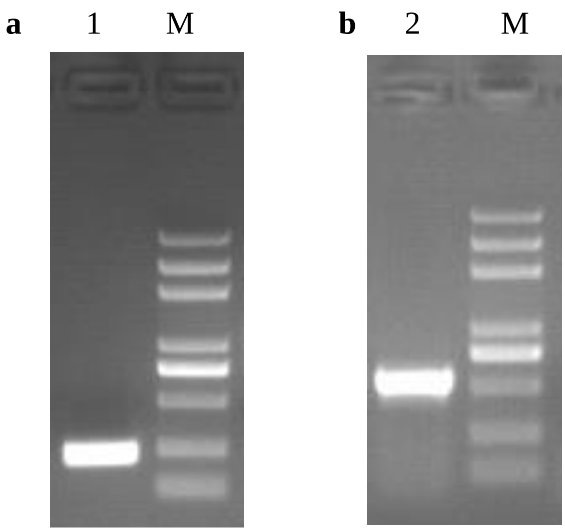

Fig. S1 5’-RACE PCR electropherograms of *niaD* (a) and *niiA* (b)

M：DL5K DNA marker (Down to up: 100, 250, 500, 750, 1000, 2000, 3000, 5000 bp)

1: 5’-RACE PCR amplification product of *niaD* (198 bp); 2: 5’-RACE PCR amplification product of *niiA* (541 bp)

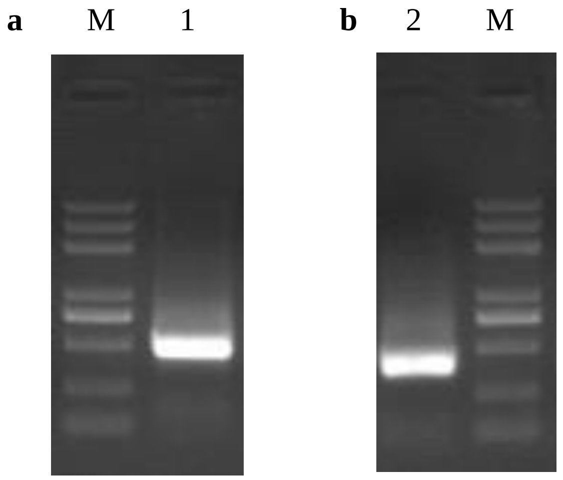

Fig. S2 3’-RACE PCR electropherograms of *niaD* (a) and *niiA* (b)

M：DL5K DNA marker (Down to up: 100, 250, 500, 750, 1000, 2000, 3000, 5000 bp)

1: 3’-RACE PCR amplification product of *niaD* (438 bp); 2: 3’-RACE PCR amplification product of *niiA* (395 bp)

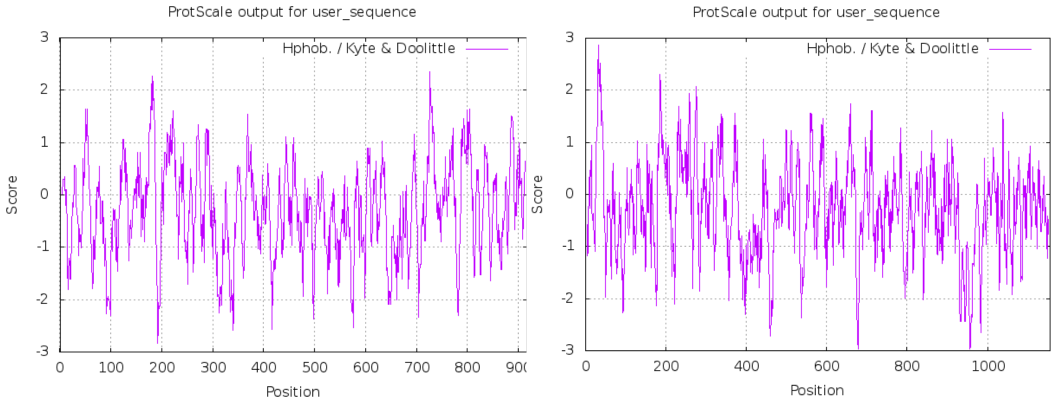

Fig. S3 The ExPASy online hydrophobic prediction of *Pisolithus* sp.1 NR (a) and *Pisolithus* sp.1 NiR (b).

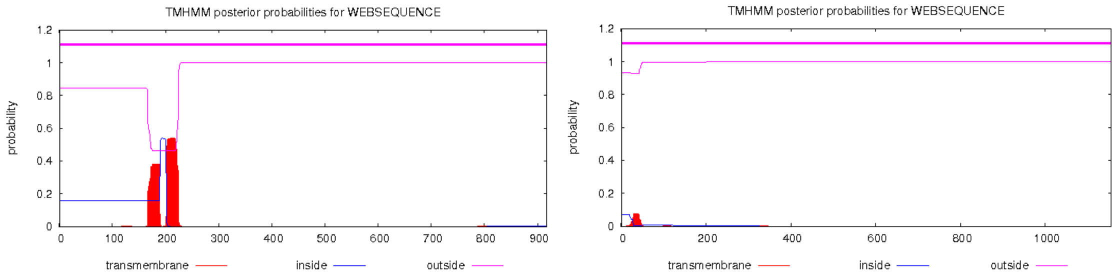

Fig. S4 The transmembrane-spanning domains predication of *Pisolithus* sp.1 NR (a) and *Pisolithus* sp.1 NiR (b) by using TMHMM software.

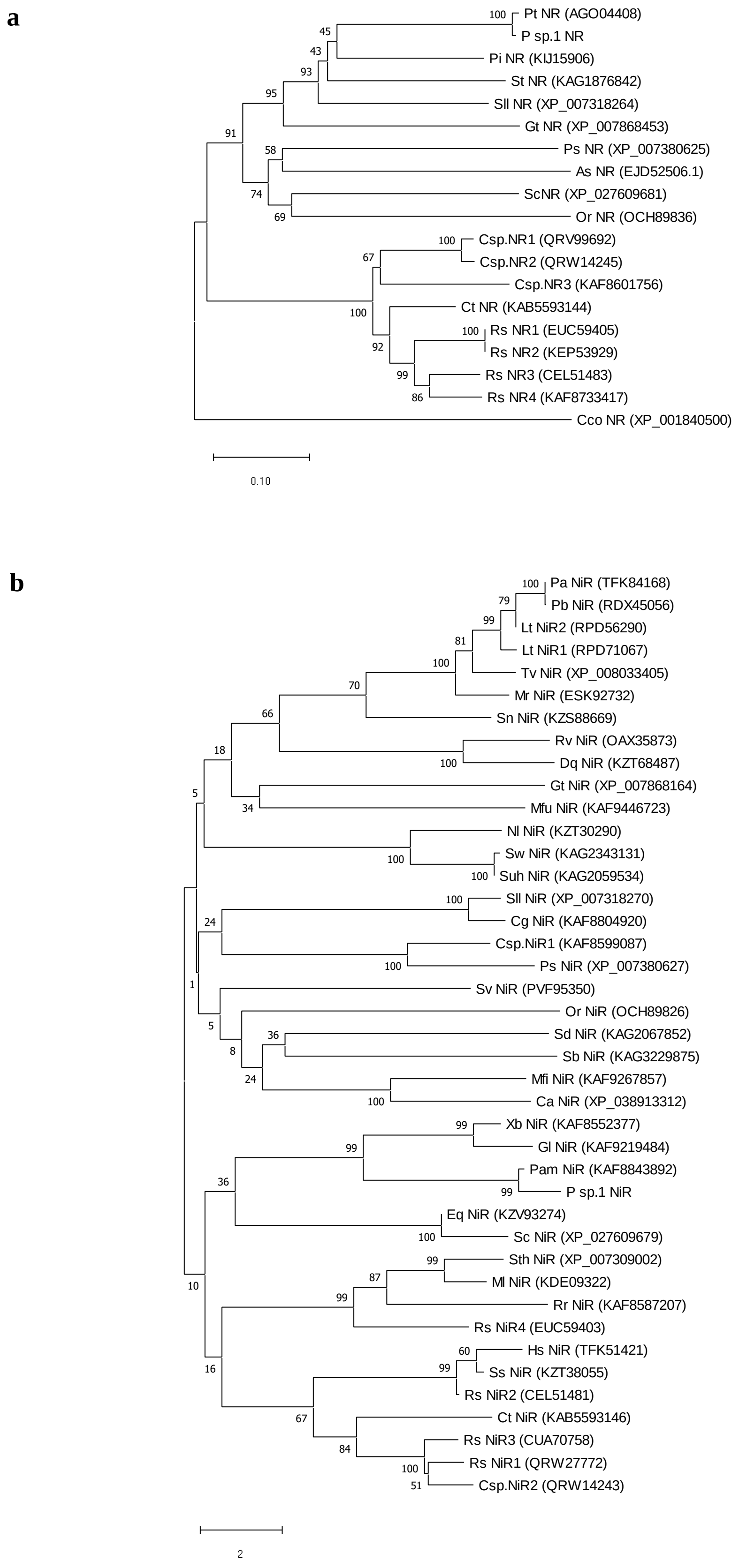

Fig. S5 Phylogenetic trees derived from NR (a) and NiR (b) amino acids sequences of *Pisolithus* sp.1 and the other EMF

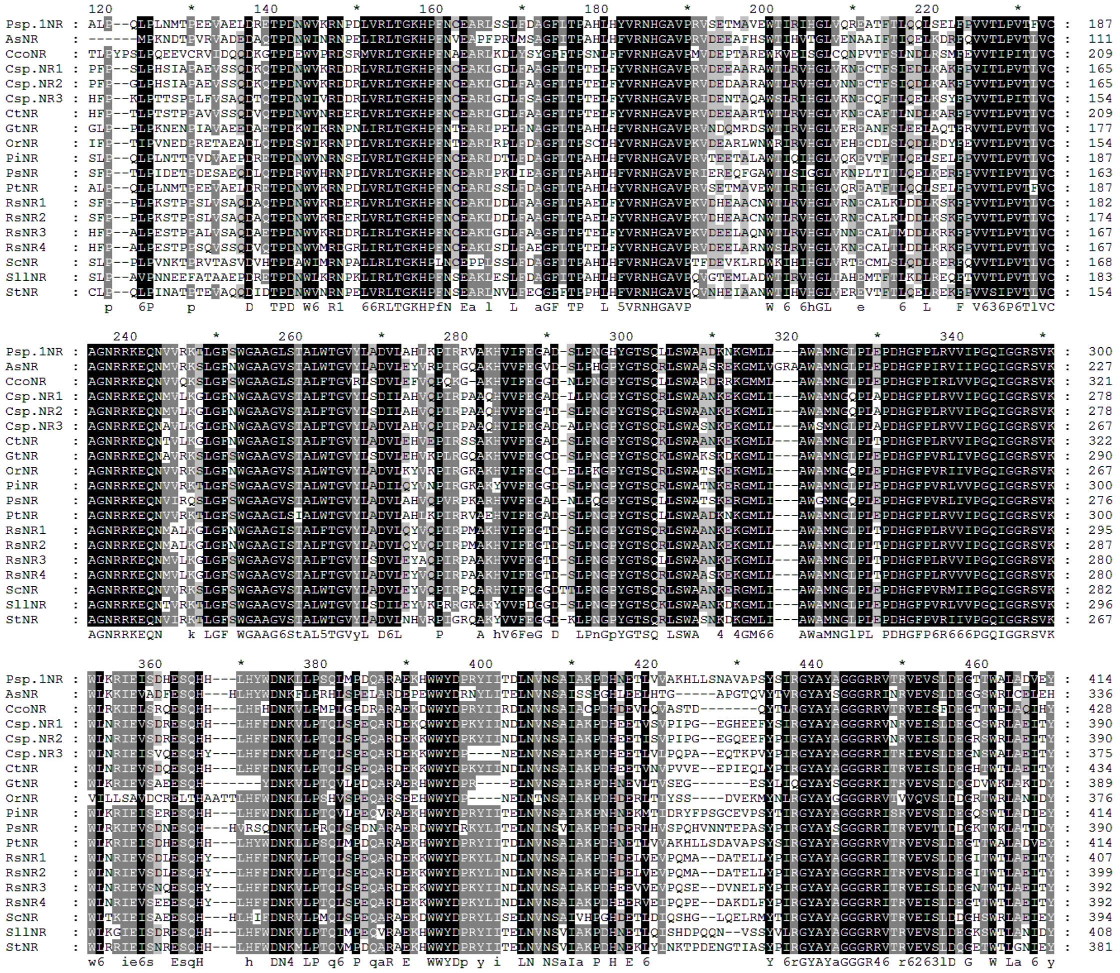

Fig. S6 Multiple sequence alignment of the molybdopterin binding domain in *niaD* encoded amino acid from *Pisolithus* sp.1 and other EMF. Accession numbers are as follows: *Pisolithus* sp.1 (Psp.1NR); *Auricularia subglabra* (AsNR), EJD52506; *Coprinopsis cinerea okayama* (CcoNR), XP_001840500; *Ceratobasidium* sp. (Csp.NR1), QRV99692; *Ceratobasidium* sp. (Csp.NR2), QRW14245; *Ceratobasidium* sp. (Csp.NR3), KAF8601756; *Ceratobasidium theobromae* (CtNR), KAB5593144; *Gloeophyllum trabeum* (GtNR), XP_007868453; *Obba rivulosa* (OrNR), OCH89836; *Paxillus involutus* (PiNR), KIJ15906; *Punctularia strigosozonata* (PsNR), XP_007380625; *Pisolithus tinctorius* (PtNR), AGO04408; *Rhizoctonia solani* (RsNR1), EUC59405; *Rhizoctonia solani* (RsNR2), KEP53929; *Rhizoctonia solani* (RsNR3), CEL51483; *Rhizoctonia solani* (RsNR4), KAF8733417; *Sparassis crispa* (ScNR), XP_027609681; *Serpula lacrymans* var. *lacrymans* (SllNR), XP_007318264; *Suillus tomentosus* (StNR), KAG1876842.

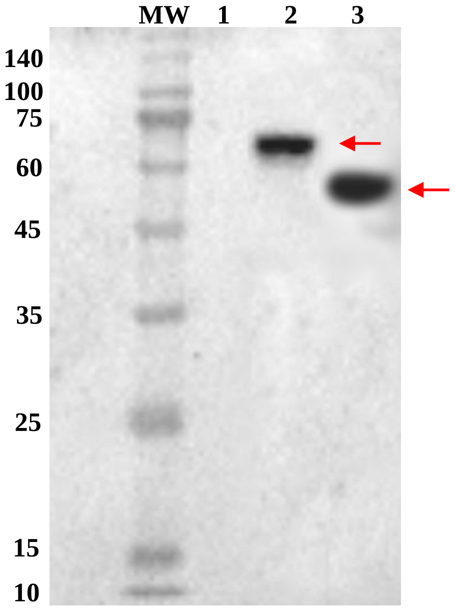

Fig. S7 Expression of *niiA* and *niaD* in *E. coli* BL21. MW, protein molecular weight standards. Lane 1, crude extracts of *E. coli* BL21/pET28b (control). Lanes 2, purified extracts of *E. coli* BL21/pET28b-*niiA*. Lanes 3, purified extracts of *E. coli* BL21/pET28b-*niaD*. The target proteins were indicated by red arrows

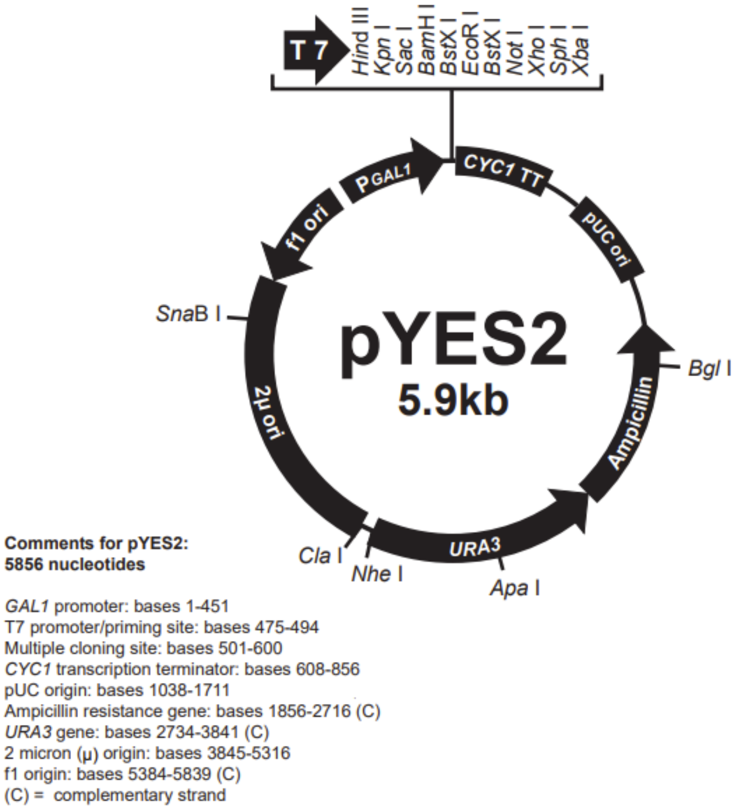

Figure. S8 The map of pYES2 yeast expression vector.

Table S1. Primers used in this study

Name	ID	Annotaion	Sequence
niaD	TRINITY_DN15759_c0_g1	Nitrate reductase	CCCAACTACAAGATATCTTAAGAATGTTTGAC<br>GAATATGTCGACTCACTGATCTTTGAACAGTC<br>TTCGTCGAGCAGAAGCAGCAGCTCGCCGGAG<br>TCAACTCTGCTGTCCACTCCTTTCACTTCCCCG<br>CCTGCGTCACCCGAACCTTCACCCGAGTCCTT<br>AACAAAGGGTGGCTTACAACTCCTTTCAGCA<br>ACATTCTTACCAGCATCTCTCCACGACGGCTC<br>TGCAAGAGATGGGCCCCCCTTACCTGAAGCCC<br>TCCCCCAATTGCCACTTAATATGACGCCGGAG<br>GAGGTTGCCGAGCTGGATCGCGAGACTCCTG<br>ACAATTGGGTAAAAAGAAATCCAGACCTCGTC<br>CGTTTGACTGGAAAACACCCTTTCAACTGCGA<br>AGCACGGCTCTCGTCGCTCTTCGATGCGGGAT<br>TCTTGACACCAGCCCATTTGCACTACGTTCGG<br>AATCATGGAGCAGTGCCTCGGGTCAGCGAGA<br>CAATGGCCGTCGAATGGACCATACGCATCCAT<br>GGCCTTGTACAGCGCGAAGCAACGTTTACCTT<br>GCAACAACTAAGCGAATTGTTCCCCGTTGTGA<br>CACTACCAGTGACGTTTGTGTGTGCCGGGAAC<br>AGAAGGAAGGAGCAAAACGTCGTTAGGAAA<br>ACCCTTGGATTCAGCTGGGGAGCTGCAGGATT<br>ATCCACCGCGCTATGGACAGGCGTCTATTTAG<br>CAGATGTCTTGGCACATCTTAAACCGATACGC<br>AGAGTGGCGAAACACGTAATCTTCGAGGGAG<br>CAGACAGCCTTCCCAACGGCCATTACGGAACT<br>TCTCAACTCCTATCTTGGGCAGCGGACAAGAA<br>CAAGGGGATGCTTCTAGCATGGGCCATGAACG<br>GCCTACCTCTCGAGCCAGACCATGGCTTTCCA<br>TTAAGGGTTGTCATCCCCGGACAGATCGGAGG<br>CCGATCCGTGAAGTGGCTAAAGCGAATCGAGA<br>TCAGTGATCATGAGAGCCAACATCATCTACATT<br>ATTGGGATAACAAATTGCTCCCAAGCCAACTC<br>ATGCCTGATCAAGCACGTGCTGAGAAGCACTG<br>GTGGTATGATCCCAGATATATCATTACAGATCT<br>AAACGTGAACAGCGCAATTGCTAAACCCGAC<br>CACAACGAGACATTGGTTGTGGCCAAGCACTT<br>ACTATCGAACGCTGTCGCCCCTTCGTACTCCAT<br>TCGCGGGTACGCTTACGCTGGCGGCGGACGA<br>CGTGTGACTCGGGTAGAGGTATCACTAGACGA<br>AGGGACAACGTGGGCCTTAGCGGATGTCGAAT<br>ACCCCGAAGATCGTTTCCGTGAGATTTGCCAC<br>GAGGACTCGACATACGGAATTCTCGACCTCTC

	TGAGAGAGATACGTCATTCTGCTGGTGTTTCT<br>GGTCCTTCGACGTGAAGTACGATGCTCTGACA<br>AAGTGTAATGCACTGGCTGTTAGGGCATCGGA<br>CGAGAGCAGCATACTTCAGCCACGAGATATGT<br>ATTGGCACTCGTTAGGCATGATGAACAACTGG<br>TGGTTCAGGGTAGCCGTGAACAAGACCGAGA<br>ACGAGAACGACATTGTTCTGCATTTCGAGCAT<br>CCGACCCTCGCTGGGACAGCAAGTGGAGGCT<br>GGATGGAACGATTGAAAACGTCAGGTCAGGA<br>CATCACGAAACCAGTATTCGGGAAGACTGGCA<br>TTGAAGAGAAACTCAATGATGTCAAACCAAA<br>AGTGGAGGAAGTACCCTTAACCAAACCAGGG<br>GTCACTCGGAAAATTACTATTGAAGAGTTGAA<br>GAACCATGATCGGGCACAGCCCTGGTTTGTCG<br>TCAACGGAGAGGTTTACGATGGAACGCCTTTT<br>TTGAACGAACATCCCGGCGGCAGCGATTCGAT<br>CACCCTTGTCGCAGGCGAGGATGCTTCCGAGG<br>ACTTCTTCGCGATCCATTCAGCGGAAGGGAAA<br>GCAAAACTGGCGCAATTCCACATCGGGACTTT<br>AGTAAAGTCAGGAACAGGAAACGCGGATCCT<br>GATGACAAAACAGCAGATGGCGAATTTCTGG<br>AGCGAGCGAAGTGGAAAGATGTCAAGCTGAC<br>AAGAAAAGTTCAAGTAAACCACGACACCTTC<br>CTCTATCGATTCGAATTACCACGGCCAGATCAA<br>CCACTTGGACTTCCTGTGGGGCAGCACGTATT<br>CGTTCGTCTCAAGAGAAAAGACACCGGCGAG<br>CTTGTCCAAAGGGCGTACACTCCTGTGACACC<br>AGAGGGAGCTGTCGGGTTCATCGAGTTATTAG<br>TCAAGTTGTACCTACCGTCCGCAGAGTTTCCG<br>GCAGGCGGCAAGATGACGACAGGATTCCATC<br>AGCTTGAAGTAGCTGACACTGTGCAACTGAA<br>GGGCCCGCTGGGATCCTTCATATGGAAGGGGC<br>TGGGCGTGGCAAATTGGAGAGGTTCCGAGAG<br>GAAAGTAAAGGAGGTTGCAATGATATGCGGTG<br>GAAGCGGTATTACGCCAATCCTCCAGGTCCTG<br>CGCAGCATCTTCCTTGACACCAGCGATACAGA<br>GACGAGAGTATGGGTGATAAGTGCGAATAGGA<br>CCGAGGAGGACATTCTCTGTCGTGCTGAACTT<br>GACCTATTGTTTGCACAACACGGCCCACATCG<br>CCTCAAGCTGCATTACGTCCTCAGTAAGGCAC<br>CAGCCACATGGGAAGGGAGTACTGGCAGAAT<br>CAACGAAGAGCTGCTGAAAGCTCACATACCG<br>GGTCCGTCCGAACACGGTATAGTCCTAGTGTG<br>CGGACCAGATCCAATGATCAACCAAGCTGTGA

			AGCCAGGACTCAAGGCCGCTGGGTGGAATAT CGAGAACCACCTCGTAGTATTCTGAAATCAAG CTTACAAGTACATGATGCTGCAGCACGAACGT GATGCACCATCTGCCTGTGTTTACTGTATAGGA GCTGTCAGTACGATTCACACTTCCATCGTTCTA CATACCATTACTGCTGAATAACCCATGGATACA ATTTGCTTAGAACACGCTGACCTTCC
niiA	TRINITY_DN 14618_c0_g1	Nitrite reductase	CTCGTTATCTCTGCGTCGTGCCTGTGACAGTG CGACAGCACTTACTAGGCGATATTCGGAGATA AGCCCCTCGACTCACCTCGGAATAGGCCAGG AACTCTGAAGGAAAATGGAGTTGATTTCTCTG ATATCTGGATATGCGACGGGGAGACATCGTTTC GTCATTTTTGCGACTCGGTTGGAGCCTTATCAT AACCGAAACGCTATCAGTCAAGTCGAAAGGC AGCTGTCCATTACACTGACCTCAACCACACTC TCCCGCCCGAACCCCCATCACACTGAGGACTG TGTCGATAGAAGTCGAACGTGCCATGATGAAC AGTACACTAGGCCAGAGTGACTGCGTTGGTGT GCCAGCATCCCATGCTTCCAAACTACTCGGGA CACCTAAGACCATTCTCGTGGTTGGACTGGGA ATGGTGGGCATTGCCTTTATTGAGAAGATACTC AACTTAGATGAGGCCAAACACTATCGGGTGG TAACCTGTGGCGAAGAAACGCACCTGGCTTAC AATCGCGTGGCTCTTACAGAATATTTTCAACAC CGCTCCGTGGAGAAGCTTTACCTTAATCAAGT GGAATGGTACGCTCGGCAGGACCCCGAGCATT TTGTTTTTTATACCGGCGAACAAGTCACATCTA TGAACACCGCTGCGCACTTCGTAAAGACGAG CAAAGGCCGTCTAATTAATTACGATTACTGCGT GCTTGCTACAGGCTCTGAGAGTACACTGCCAC CGTATATCCCCCCGGAACGTGTCGCGCAGACC AAGGGTGTTTTCGTCTATCGAAACATTTCGGA CCTCGATAAGATCTTGAGTTACTCTGAAGAAA ATCATGTCAGAGGCGGCCGGGCTGTGGTTGTT GGCGGGGGTCTACTTGGTCTTGAAGCTGCCAA AGCTGTCTTTGATCTAGAAACCATCGGGAAAG TCACTATAATCAATCGCCAGGCATATCCTCTAT CTCGTCAGCTTGACGACGAGGGTGGCGAGAT TGTCCTGCGTTGCATTGAGGCCATGGGAGTTG AAGTCCTCACAAAGACGTCTGTAACGAGTCTC GTGACCACTCCGGAGGGCGTCTTGACCGGAC TTGTCCTATCAAACGATGATCAATTGGACGCC GAGATGGTTGTTTATGCAATCGGGATTTCCCCT CGCGATGATTTAGCAAGGAAAGCAGGGTTGA

	AATGTGGGGTGAGAGGTGGAATCACTGTGGA<br>CGATTACCTAAGGACAAGTATTCCCGATATCTA<br>CGCGATCGGAGAGTGTGCCAGCTGGCGAGAG<br>AATACCTTCGGTCTTATTGGCCCAGGAGTCGA<br>AATGGCGGACATCCTGGCCTTCAACCTTACTC<br>AAGTGCAGACCGACGTCGGAGGTTTTAAACCT<br>AGACAAATGAACATGCCTGATTTGTCAACGAA<br>GCTGAAGCTCATGGGTGTTGATGTTGCTTCTT<br>TTGGCGACTACTTTCTAGATAAGCGAGTACCC<br>CGGGGCACGGCTACCAGAAGTCGGAAGGTCG<br>AAGATACCCAAGCTGACAAGCTTTCACGTGCC<br>GGATCTGCTCAAGACGGAAAGCCGGCTTGGG<br>AAATTAAGATCGACGACTCCAATGCCTCGACA<br>ACCCCAGCATCCAACAAGCAGCACATACTGA<br>AATCGGGTAGCGTCTCTCTGCAGAAGGTTTCC<br>TCAATTACGACCACGAGCGAAGATAATTCGAA<br>GAGACACGGCTCCGGTTCTAATGAACCTATAG<br>AATGTCTGACCTATAGGGATCCATTTTCTTTTG<br>TGTATAAAAAGTACATCTTCACTGCAGACGGC<br>AAACATCTGCTAGGCGGGATGATGGTTGGGGA<br>CACCTCGGATTATGTCAAACTTATTAGCTTGGT<br>CAAGAAAAAGAAAGCTATCGATGTGCCGCCAT<br>CACAATTCATTGTTGGAGTCGGAAGGAAAGG<br>CGAGGACGATGGCGCCGACCTGGATGATGAC<br>ACTCAAGTTTGCAGTTGTCATAACGTGACGA<br>AGGGTGCTATTGTGGCATGTGTCAAAGAAGGC<br>ATCAGCGGCATGGAGGACTTGAAGTGCAAAA<br>CCAAAGTTGGAACTGGCTGCGGCGGTTGTATG<br>CCCTTAGTCACCAATCTCTTCAAGTCTGAAAT<br>GAAGAAAGCTGGACACGCTGTGTCTAACAAC<br>CTTTGCACACACTTCACCATGTCTCGTCAGGA<br>ACTCTTCACTGTCATTAAACTACGGAAGTTGA<br>GGACGTTTCCTGAGGTCATGCAATCTGCGGGA<br>GCTAAGCCAGACTCGGTTGGCTGCGAGTTGTG<br>CAGGCCTGCAGTTGCATCTATCCTATCTTCAAT<br>GTACAACGAACTCGTTGTCGCCCCATCACATC<br>ATTCTAACCAAGACACCAATGACAGGTGCCTG<br>GCAAACATACAGAGAGATGGTACATTCTCCGT<br>GGTGCCGCGAGTTGCTGGAGGCGAGATCACT<br>CCAGACAAGTTGATTGTTCTTGGACAAGTTGC<br>AAAGAAATACAAATTATACACGAAGATAACGG<br>GAGGACAACGCATTGATTTGTTTGGTGCGCAA<br>AAGCAAGACCTCCCAGACATTTGGGAAGAAC<br>TAGTTAATGCTGGATTTGAGAGTGGCCATGCAT

	ATGGCAAAGCGTTGCGTACCGTCAAATCCTGC<br>GTGGGCACGACGTGGTGCAGATACGGTGTTG<br>GAGATTCAGTCGGAATGGCGATCCGTCTAGAG<br>GAACGTTACAAAGGTATACGCTCGCCTCATAA<br>ACTTAAAGGCGGGGTCAGTGGTTGCACCAGA<br>GAGTGCGCAGAAGCGCAGTCAAAGGATTTTG<br>GTTTGATCGCCACAGACAAGGGCTGGAACATC<br>TTCTTGGCTGGGAACGGCGGCACGAACCCTC<br>GCCATGCCACGCTGTTCGCGAAGGATGTACCG<br>CCTTCGAAAGTCATACGAATTCTTGACCGTTT<br>CCTCATGTACTATATTCGAACAGCAGACAAGC<br>TTATGCGCACTGCGCGCTGGGTCGAACAGTTT<br>GAGGGAGGAATCGAGAGACTTCAAAAGATCT<br>TGTTAGACGATGAACTCGGGATTTGTGATGAG<br>CTCGAGGCTGAGATGGCTACCTTGGTCGGCA<br>CGTACCATGACGAGTGGAGTGTAGTAGTCAAG<br>GATCCTGCCAGGCGGCGGCAGTTTCGCCAATT<br>TGTAAACACAGATGAGCGTCAACAGCAGGTA<br>GAGATTATAAAAGAGCGCGAACAGCAGCGTC<br>CCGCCGATTGGCCCAAGCAATTTCCCCCGGCG<br>AAATTCCATTCCTCCGCCATTGGAACGCCCAA<br>GTCCGAGTGGAAGTGGCGCAAGTTGGCCACA<br>GTCCAAGACTTAGAGCTTTCTGATACTACCTCT<br>TCTGTAGCTGTGAAATACGGAGACTCTCAATT<br>GGCCATTTTCCACGTACCAAAGAAGGGTTTCT<br>TCGCTACTCAGCAGATGTGCCCTCACAAACGT<br>GCATTTGTGCTTGACCATGGTATCGTCGGCGA<br>CGATAAACAAGGCGACATTTATGTTTCTTGTCC<br>GCTACACAAGCGTAACTTCCGCCTTAGCGACG<br>GTACCTGCCTGAACGACATGCAGTATTCAATC<br>ATTTCATTCGACATTAGACAAGAAGGGGACGA<br>CCTGCAGCTCCTGCTTCCCGAGCCTGAAGAAC<br>TAGATGCTGTCATAGGCACCAGCAAATGGATG<br>ATCAAGCAGGCCACGGCCGAGATTCTATCTCG<br>CGGTGGTGGCCAAGGTATCGAGATCGCAGCG<br>GACCACTTAGATGGGCCGCCAGCTGCCACTGG<br>TGCAGGATGTAATGGAGATTCTTGTGGCGACT<br>CCACTTTAGATTGGTGAACCTGTTGACCAGAC<br>CTCAAATATACAGTACCCCCTACAGCGATCGGT<br>TTAGAGATATATATATGCCCCATATTTGATGTAT<br>CTGTTTTGTACAGTATCGTATGCATTGAGCAGT<br>CAGATTGTAAAGCCATTCGCGCAGATGTCAAA<br>TTG

Table S2. Gene IDs and accession numbers of the mycorrhizal fungal NRs used in Fig. S5

Species	Gen ID	Accession number	References
Auricularia subglabra	AsNR	EJD52506	https://www.ncbi.nlm.nih.gov/protein/EJD52506.1/
Coprinopsis cinerea okayama	CcoNR	XP_001840500	https://www.ncbi.nlm.nih.gov/protein/XP_001840500.2/
Ceratobasidium sp.	Csp.NR1	QRV99692	https://www.ncbi.nlm.nih.gov/protein/QRV99692.1/
Ceratobasidium sp.	Csp.NR2	QRW14245	https://www.ncbi.nlm.nih.gov/protein/QRW14245.1/
Ceratobasidium sp.	Csp.NR3	KAF8601756	https://www.ncbi.nlm.nih.gov/protein/KAF8601756.1/
Ceratobasidium theobromae	CtNR	KAB5593144	https://www.ncbi.nlm.nih.gov/protein/KAB5593144.1/
Gloeophyllum trabeum	GtNR	XP_007868453	https://www.ncbi.nlm.nih.gov/protein/XP_007868453.1/
Obba rivulosa	OrNR	OCH89836	https://www.ncbi.nlm.nih.gov/protein/OCH89836.1/
Paxillus involutus	PiNR	KIJ15906	https://www.ncbi.nlm.nih.gov/protein/KIJ15906.1/
Punctularia strigosozonata	PsNR	XP_007380625	https://www.ncbi.nlm.nih.gov/protein/XP_007380625.1/
Pisolithus tinctorius	PtNR	AGO04408	https://www.ncbi.nlm.nih.gov/protein/AGO04408.1/
Rhizoctonia solani	RsNR1	EUC59405	https://www.ncbi.nlm.nih.gov/protein/EUC59405.1/
Rhizoctonia solani	RsNR2	KEP53929	https://www.ncbi.nlm.nih.gov/protein/KEP53929.1/
Rhizoctonia solani	RsNR3	CEL51483	https://www.ncbi.nlm.nih.gov/protein/C

			EL51483.1/
Rhizoctonia solani	RsNR4	KAF8733417	https://www.ncbi.nlm.nih.gov/protein/KAF8733417.1/
Sparassis crispa	ScNR	XP_027609681	https://www.ncbi.nlm.nih.gov/protein/XP_027609681.1/
Serpula lacrymans var. *lacrymans*	SllNR	XP_007318264	https://www.ncbi.nlm.nih.gov/protein/XP_007318264.1/
Suillus tomentosus	StNR	KAG1876842	https://www.ncbi.nlm.nih.gov/protein/KAG1876842.1/

Table S3. Gene IDs and accession numbers of the mycorrhizal fungal NiRs used in Fig. S6

Species	Gen ID	Accession number	References
Cantharellus anzutake	CaNiR	XP_038913312	https://www.ncbi.nlm.nih.gov/protein/XP_038913312.1/
Cortinarius glaucopus	CgNiR	KAF8804920	https://www.ncbi.nlm.nih.gov/protein/KAF8804920.1/
Ceratobasidium sp.	Csp.NiR1	KAF8599087	https://www.ncbi.nlm.nih.gov/protein/KAF8599087.1/
Ceratobasidium sp.	Csp.NiR2	QRW14243	https://www.ncbi.nlm.nih.gov/protein/QRW14243.1/
Ceratobasidium theobromae	CtNiR	KAB5593146	https://www.ncbi.nlm.nih.gov/protein/KAB5593146.1/
Daedalea quercina	DqNiR	KZT68487	https://www.ncbi.nlm.nih.gov/protein/KZT68487.1/
Exidia qlandulosa	EqNiR	KZV93274	https://www.ncbi.nlm.nih.gov/protein/KZV93274.1/
Gyrodon lividus	GlNiR	KAF9219484	https://www.ncbi.nlm.nih.gov/protein/KAF9219484.1/
Gloeophyllum trabeum	GtNiR	XP_007868164	https://www.ncbi.nlm.nih.gov/protein/XP_007868164.1/
Heliocybe sulcata	HsNiR	TFK51421	https://www.ncbi.nlm.nih.gov/protein/TFK51421.1/
Lentinus tigrinus	LtNiR-1	RPD71067	https://www.ncbi.nlm.nih.gov/protein/RPD71067.1/
Lentinus tigrinus	LtNiR-2	RPD56290	https://www.ncbi.nlm.nih.gov/protein/RPD56290.1/
Marasmius fiardii	MfiNiR	KAF9267857	https://www.ncbi.nlm.nih.gov/protein/KAF9267857.1/

Macrolepiota fuliginosa	MfuNiR	KAF9446723	https://www.ncbi.nlm.nih.gov/protein/KAF9446723.1/
Microbotryum lychnidis-dioicae	MlNiR	KDE09322	https://www.ncbi.nlm.nih.gov/protein/KDE09322.1/
Moniliophthora roreri	MrNiR	ESK92732	https://www.ncbi.nlm.nih.gov/protein/ESK92732.1/
Neolentinus lepideus	NlNiR	KZT30290	https://www.ncbi.nlm.nih.gov/protein/KZT30290.1/
Obba rivulosa	OrNiR	OCH89826	https://www.ncbi.nlm.nih.gov/protein/OCH89826.1/
Polyporus arcularius	PaNiR	TFK84168	https://www.ncbi.nlm.nih.gov/protein/TFK84168.1/
Paxillus ammoniavirescens	PamNiR	KAF8843892	https://www.ncbi.nlm.nih.gov/protein/KAF8843892.1/
Polyporus brumalis	PbNiR	RDX45056	https://www.ncbi.nlm.nih.gov/protein/RDX45056.1/
Punctularia strigosozonata	PsNiR	XP_007380627	https://www.ncbi.nlm.nih.gov/protein/XP_007380627.1/
Ramaria rubella	RrNiR	KAF8587207	https://www.ncbi.nlm.nih.gov/protein/KAF8587207.1/
Rhizoctonia solani	RsNiR-1	QRW27772	https://www.ncbi.nlm.nih.gov/protein/QRW27772.1/
Rhizoctonia solani	RsNiR-2	CEL51481	https://www.ncbi.nlm.nih.gov/protein/CEL51481.1/
Rhizoctonia solani	RsNiR-3	CUA70758	https://www.ncbi.nlm.nih.gov/protein/CUA70758.1/
Rhizoctonia solani	RsNiR-4	EUC59403	https://www.ncbi.nlm.nih.gov/protein/EUC59403.1/
Rhizopogon vinicolor	RvNiR	OAX35873	https://www.ncbi.nlm.nih.gov/protein/OAX35

			873.1/
Suillus brevipes	SbNiR	KAG3229875	https://www.ncbi.nlm.nih.gov/protein/KAG3229875.1/
Sparassis crispa	ScNiR	XP_027609679	https://www.ncbi.nlm.nih.gov/protein/XP_027609679.1/
Suillus decipiens	SdNiR	KAG2067852	https://www.ncbi.nlm.nih.gov/protein/KAG2067852.1/
Serpula lacrymans var. *lacrymans*	SllNiR	XP_007318270	https://www.ncbi.nlm.nih.gov/protein/XP_007318270.1/
Sistotremastrum niveocremeum	SnNiR	KZS88669	https://www.ncbi.nlm.nih.gov/protein/KZS88669.1/
Sistotremastrum suecicum	SsNiR	KZT38055	https://www.ncbi.nlm.nih.gov/protein/KZT38055.1/
Stereum hirsutum	SthNiR	XP_007309002	https://www.ncbi.nlm.nih.gov/protein/XP_007309002.1/
Suillus hirtellus	SuhNiR	KAG2059534	https://www.ncbi.nlm.nih.gov/protein/KAG2059534.1/
Serendipita vermifera	SvNiR	PVF95350	https://www.ncbi.nlm.nih.gov/protein/PVF95350.1/
Suillus weaverae	SwNiR	KAG2343131	https://www.ncbi.nlm.nih.gov/protein/KAG2343131.1/
Trametes versicolor	TvNiR	XP_008033405	https://www.ncbi.nlm.nih.gov/protein/XP_008033405.1/
Xerocomus badius	XbNiR	KAF8552377	https://www.ncbi.nlm.nih.gov/protein/KAF8552377.1/
